# A systematic review on cough sound analysis for Covid-19 diagnosis and screening: is my cough sound COVID-19?

**DOI:** 10.7717/peerj-cs.958

**Published:** 2022-04-25

**Authors:** KC Santosh, Nicholas Rasmussen, Muntasir Mamun, Sunil Aryal

**Affiliations:** 1 2AI: Applied Artificial Intelligence Lab, Computer Science, University of South Dakota, Vermiillion, South Dakota, United States; 2 School of Information Technology, Deakin University, Victoria, Australia

**Keywords:** Covid-19, Cough sound, Diagnosis, Public healthcare, AI, Machine learning

## Abstract

For COVID-19, the need for robust, inexpensive, and accessible screening becomes critical. Even though symptoms present differently, cough is still taken as one of the primary symptoms in severe and non-severe infections alike. For mass screening in resource-constrained regions, artificial intelligence (AI)-guided tools have progressively contributed to detect/screen COVID-19 infections using cough sounds. Therefore, in this article, we review state-of-the-art works in both years 2020 and 2021 by considering AI-guided tools to analyze cough sound for COVID-19 screening primarily based on machine learning algorithms. In our study, we used PubMed central repository and Web of Science with key words: (Cough OR Cough Sounds OR Speech) AND (Machine learning OR Deep learning OR Artificial intelligence) AND (COVID-19 OR Coronavirus). For better meta-analysis, we screened for appropriate dataset (size and source), algorithmic factors (both shallow learning and deep learning models) and corresponding performance scores. Further, in order not to miss up-to-date experimental research-based articles, we also included articles outside of PubMed and Web of Science, but pre-print articles were strictly avoided as they are not peer-reviewed.

## Introduction

**Figure 1:**
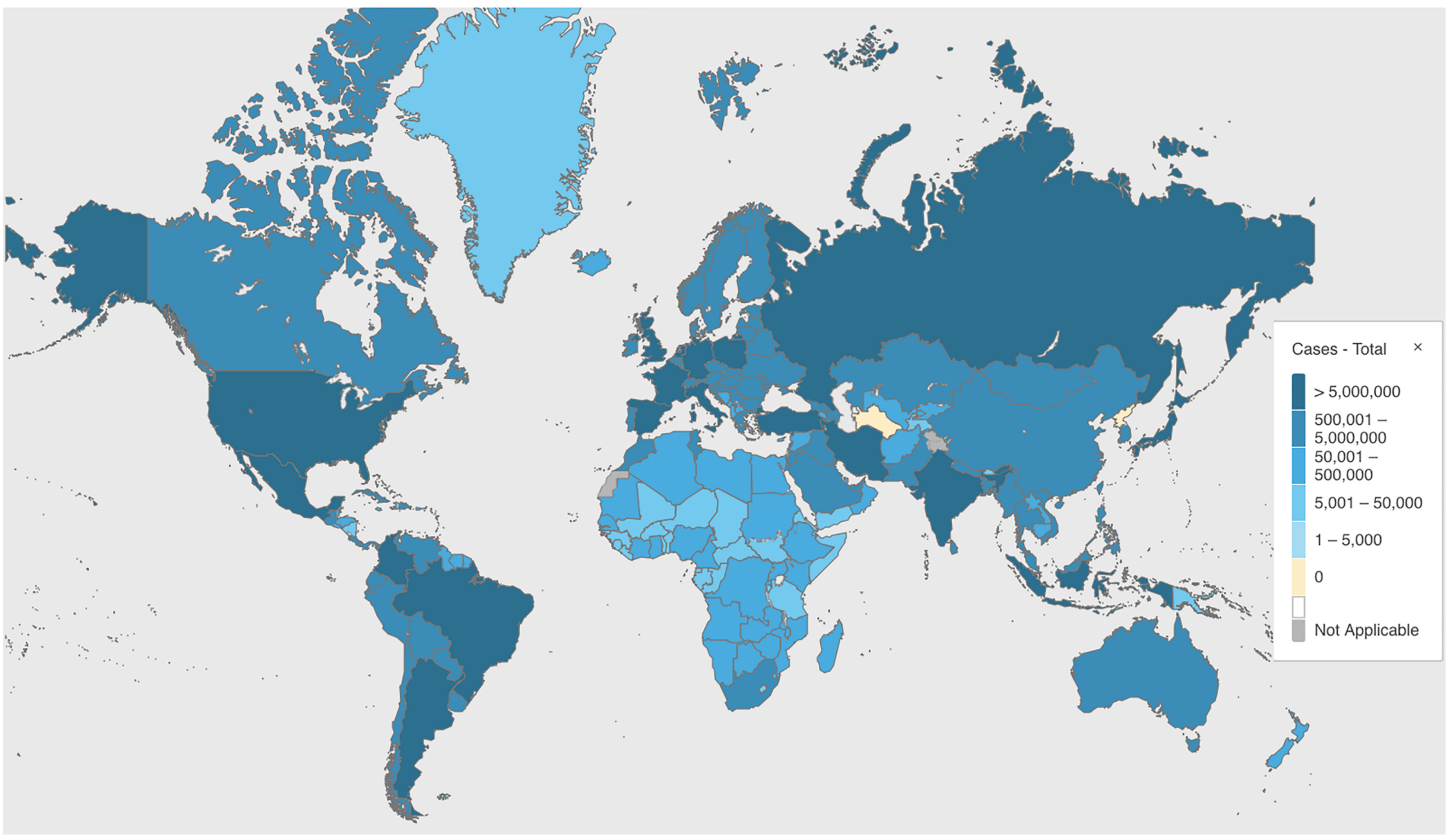
Globally, as of 4:31pm CET, 8 March 2022, there have been 446,511,318 confirmedcases of COVID-19, including 6,004,421 deaths (source: https://covid19.who.int).

COVID-19 has ravaged the world since the World Health Organization (WHO) declared it a global health emergency on January 30, 2020. There are 446,511,318 total confirmed cases and 6,004,421 deaths worldwide as of March 8, 2022 (see Fig. 1) (WHO coronavirus (COVID-19) dashboard. https://covid19.who.int/). Despite all our best efforts, the human cost in terms of person-hours spent and lives lost to this virus is extensive. Although there are multiple ways to fight the virus, the greatest hope for ending the pandemic is vaccines, of which 10,704,043,684 vaccine doses (as of March 6, 2022) have been distributed globally (Ahmed et al., 2020). However, if we rely on vaccines alone, there will be many more deaths before humanity can put this pandemic behind it. Therefore, how else can we fight this virus other than the suggested preventative measures, like washing our hands and wearing masks? Other than diagnosis, screening tests will be one of the most integral parts of containing this virus completely, especially in resource-constrained regions across the world, where vaccines are unavailable or untrusted because of general vaccine skepticism within a community.

Cough is used to diagnose and predict many diseases to help clinicians better serve patients; some of these diseases include but are not limited to pneumonia and asthma. Multiple studies have recently used artificial intelligence (AI)-guided tools to help classify different coughs and help diagnose or predict certain diseases. For example, Nemati et al. (2020) conducted a study to help classify the severity of chronic obstructive pulmonary disease by measuring the airflow and volume of patients breathing with spirometry tests. Nakamori et al. (2020) showed that using a simplified cough test, they can predict the risk of patients with acute stroke getting pneumonia. Another study conducted by Nemati et al. (2020) used AI-guided tools to detect whether a specific cough was ‘dry’ or ‘wet,’ which would help establish the presence of sputum. In Rudraraju et al. (2020), a study also found that AI-guided tools can classify obstructive *vs* restrictive lung problems. Lastly, Han et al. (2020) used cough and other types of valuable data to estimate the severity level of illness. These previous five examples show that different cough metrics can get leveraged in multiple ways, with and without AI-guided tools, to help diagnose and predict diseases in humans. However, because cough has helped diagnose many other diseases, AI-guided tools that leverage cough sound samples must also take into consideration the works involved with diagnosing other diseases to more accurately screen COVID-19 (Imran et al., 2020). Furthermore, mel-frequency cepstral coefficients (MFCCs) processing helps COVID-19 screening because authors found high similarity in MFCCs between different COVID-19 cough and breathing sounds (Alsabek, Shahin & Hassan, 2020). Although this is only one cough feature that most robust AI-powered algorithms use, it is a good case-in-point for the research that we will explore in this article. Based on the features like MFCCs extracted from the audio recording, AI-guided tools can help detect a cough and use this process to screen COVID-19 patients (Chen et al., 2021; Miranda, Diacon & Niesler, 2019; Chuma & Iano, 2021). More often, accuracy for basic cough detectors is generally greater than 95%. While reviewing state-of-the-art works, we consider whether AI-guided tools are reproducible (with external validation) for COVID-19 diagnosis—‘is my cough COVID-19?’ (Topol, 2020).

A brief clinical analysis of the symptom cough in COVID-19 patients reveals many factors to consider related to AI-guided tools. The first factor to consider is that there is no international definition of the symptom of cough (Morice, 2007). This fact shows that diagnosis of the symptom cough is subjective, and clinician reporting of cough can be inaccurate, and it is even more inaccurate in the general population (Donnelly & Everard, 2019; Morice, 2007). The following factors should also be get considered: socio-demographics, the severity of the illness, the patient’s age, and temporal considerations. Multiple socio-demographic studies on COVID-19 show that cough is ubiquitous in terms of the presentation rate of the symptom between different populations such as African-Americans and Latinos (Gayam et al., 2020; Weng et al., 2020). Furthermore, these studies show that there are differences in the presentation of other symptoms regarding race. Next, multiple studies regarding the severity level of the illness show little difference between mild, moderate, severe, and critical infections (Lapostolle et al., 2020; Wang et al., 2020; Vaughan et al., 2021; Matangila et al., 2020; Yang et al., 2020). However, there are a few studies, out of many, that show a difference (Fontana, 2007). Moreover, patients in the mild to moderate categories generalize well to the population at large because most COVID-19 infections will fall into this category (Yang et al., 2020). Age category is also an essential factor to consider and studies show there is little to no difference between age groups for the presentation rate of the cough symptom (Brendish et al., 2020; Cattelan et al., 2020; Guan et al., 2020; Morice, 2007). Lastly, considering the temporal aspects of cough in COVID-19 infections, we see a critical aspect that shows that cough is a reliable metric. That aspect is, the symptom of cough lasts longer and at a higher rate than all other symptoms (Faezipour & Abuzneid, 2020). This study analyzed a relatively small number of patients; however, if this study can be confirmed, it shows that cough as a screening metric may help catch an infection in the later stages. Overall, based on these studies, the cough presentation rate is high relative to all symptoms and can be considered the second most common symptom after fever.

This paper systematically reviews AI-guided tools that are used to analyze cough sound for COVID-19 screening. To avoid possible confusion between diagnostic tests and screening tests, a diagnostic test aims to establish the presence/absence of the disease, while a screening test is to detect potential disease indicators. In short, we systematically review screening tests with the use of AI-guided tools. AI-guided tools rely on fully observed clinically diagnosed data (cough sounds, in our case) and are considered one of the fastest and most accurate tools in classifying/screening COVID-19 (Imran et al., 2020; Ahmed et al., 2020). Other data, such as respiratory and breathing sounds, can also be considered for screening (Hassan, Shahin & Alsabek, 2020), but our study is limited to analyzing cough sounds because it is one of the most widespread symptoms that can easily get screened with low-cost equipment for many people in a short period of time.

The remainder of the paper is organized as follows. “Survey Methodology” presents our review methodology (inclusion criteria of the published research articles). It mainly includes AI-guided tools for COVID-19 screening using cough sounds in both crowd-sourced and laboratory confirmed data (“AI-guided tools for COVID-19 screening using cough sounds”). In addition, in “Research articles outside of PubMed and Web of Science”, we also review important findings outside PubMed and Web of Science. These sections get followed by discussion in “Discussion”. “Conclusion” concludes our study.

## Survey methodology

**Figure 2:**
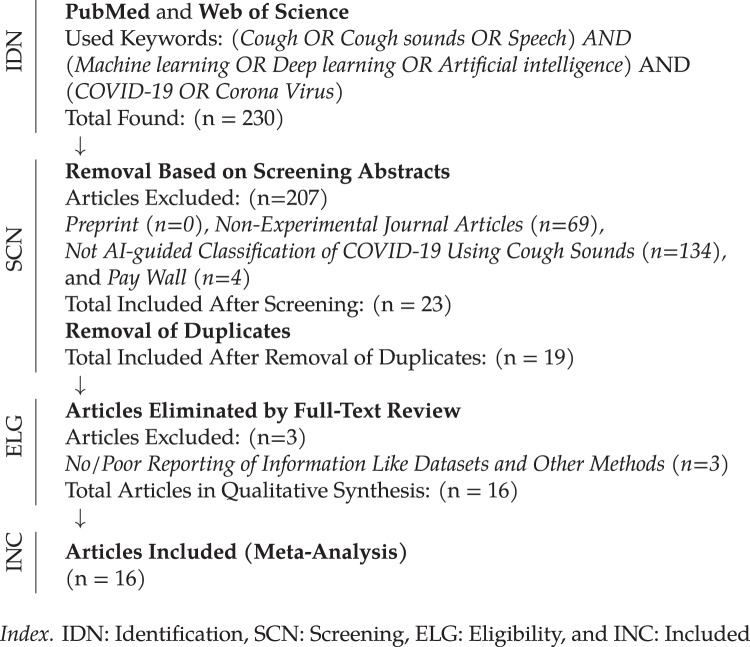
Workflow representing different phases of the systematic review (source: PRISMAcriteria (Liberati et al., 2009)).

An essential aspect of any review article is how the information was collected (McDonagh et al., 2013). For our systematic review, we follow a workflow representing different phases of systematic review, where it primarily includes identification, screening, eligibility and included criteria as shown in Fig. 2. In our study, we used PubMed central repository and Web of Science, and selected key words are (*Cough* OR *Cough Sounds* OR *Speech*) AND (*Machine learning* OR *Deep learning* OR *Artificial intelligence*) AND (*COVID-19* OR *Coronavirus*). In our screening, duplicate items were removed, and experiment-based papers are included.

For better meta-analysis, we screened for appropriate dataset (size and source), algorithmic factors (both, shallow learning and deep learning models), and corresponding performance scores. For meta-analysis, we included experimental-based research articles. Pre-print articles (*e.g*., ArXiv, medRxiv and TechRxiv) were strictly avoided as they are not peer-reviewed.

### AI-guided tools for COVID-19 screening using cough sounds

**Figure 3:**
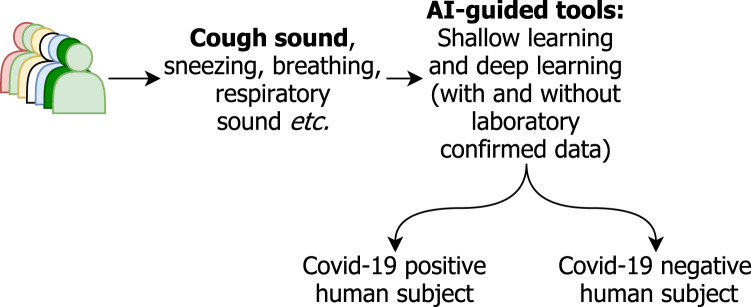
AI-guided tools for COVID-19 screening using cough sound. COVID-19 positive human subjects are classified based on both, shallow and deep learning models. Even though multiple data types can be considered, in this study, we are focusing on cough sound.

AI has contributed a lot in healthcare and integrating speech/audio processing tools is no exception (Santosh, 2019; Mukherjee et al., 2021; Mukherjee, Salam & Santosh, 2021). We review state-of-the-art works on COVID-19 screening through cough sounds. Not only that, but we also address other data types such as sneezing, respiratory, speech, throat clearing, wheezing, and breathing. For a better understanding, Fig. 3 provides a workflow of how AI-guided tools are commonly employed. It takes cough sound data as an input, extracts features and differentiates COVID-19 positive human subjects from non-COVID ones.

It is possible to develop AI-guided audio/speech processing tools/techniques that extract and leverage acoustic biomarker features to pre-screen COVID-19 recordings from cough. Before developing detailed information, let us discuss a few cases where use of biomarkers and smartphone-based tools/techniques can be seen. Experts proposed a speech and signal processing approach to analyze COVID-19 (in both cases: asymptomatic and symptomatic). A dataset with a complexity of neuromotor coordination across speech subsystems that involve respiration, phonation, and articulation, encouraged by the distinct nature of COVID-19’s lower *vs* upper respiratory tract inflammation, helps detect COVID-19 in asymptomatic and symptomatic patients (Quatieri, Talkar & Palmer, 2020). These biomarker features can get leveraged with AI-guided tools to have a significant effect to increase forced cough COVID-19 detection accuracy (Laguarta, Hueto & Subirana, 2020). Similarly, smartphone-based self-testing of COVID-19 using breathing sounds and their implications to find breathing complications by comparing specific acoustic signal patterns has also been gotten observed in this review. In Faezipour & Abuzneid (2020), the authors suggest their opinion for using advanced signal processing in tandem with new deep machine learning and pattern recognition techniques on smartphone technology.

In Tables 1 and 2, we organize the results to contrast studies with and without laboratory confirmation of their datasets for better understanding. Not limited to cough sounds, other sources of data, such as speech, breathing and respiratory sounds, are considered in some studies (see Fig. 3).

**Table 1:** Cough sounds (including other data type) for COVID-19 screening performance in terms of Accuracy (ACC), Area Under the Curve (AUC), Sensitivity (SEN) and Specificity (SPEC) on ‘no laboratory confirmed data’.

		Performance			
Authors (year)	Data type (sample size)	ACC	AUC	SEN	SPEC
Dash et al. (2021)	Cough sounds (5,130)	0.86	0.50	–	–
Mouawad, Dubnov & Dubnov (2021)	Cough sounds (1,927)	0.91	0.84	–	–
	Speech (1,488)	0.89	0.86	–	–
Shimon et al. (2021)	Cough sounds (1,296)	0.74	0.60	0.90	0.35
Despotovic et al. (2021)	Cough sounds (496)	0.88	–	0.87	0.89
Melek (2021)	Cough sounds (180)	0.98	0.98	0.97	1.00
Mohammed et al. (2021)	Cough sounds (1,276)	0.77	0.77	0.71	–
Gokcen et al. (2021)	Cough (822)	0.79	–	0.75	–
Feng et al. (2021)	Cough sounds (1,440)	0.81	0.79	–	–
Coppock et al. (2021)	Cough sounds (517)	–	0.84	–	–
Lella & Pja (2021)	Multiple data types* (6,000)	0.95	–	–	–
Pahar et al. (2021)	Cough sounds (1,171)	0.95	–	0.93	0.98
Laguarta, Hueto & Subirana (2020)	Cough Sounds (5,320)	0.97	0.97	0.98	0.94
Jayachitra et al. (2021)	Cough Sounds (601)	0.97	–	0.94	–

**Note:**

* Multiple data types, including cough sound.

**Table 2:** Cough sounds (including other data type) for COVID-19 screening performance in terms of Accuracy (ACC), Area Under the Curve (AUC), Sensitivity (SEN) and Specificity (SPEC) on ‘laboratory confirmed data’.

		Performance			
Authors (year)	Data (sample size)	ACC	AUC	SEN	SPEC
Imran et al. (2020)	Cough sounds (543)	0.93	–	0.94	0.91
Pinkas et al. (2020)	Complete recordings* (292)	0.79	–	0.79	–
Lonini et al. (2021)	Multiple data types* (288)	–	0.94	–	–

**Note:**

* Multiple data types, including cough sound.

#### Studies on non laboratory confirmed datasets

In Table 1, we summarize work in accordance with how features were extracted (shallow learning or deep learning). In other words, we consider handcrafted features (with typical machine learning classifiers) and deep learning algorithms to detect cough sounds in COVID-19 patients. Regardless of how samples were collected, we start with popular features, such as MFCCs, typical machine learning classifiers, and end with deep features. Table 1 follows the order of the description (provided below).

• Handcrafted features (shallow learning): MFCCs contributed a lot in audio/speech processing. In Dash et al. (2021), the authors used the MFCCs of speech signals. From the Coswara dataset, designated database-1, the authors used 570 participants, and each participant contributed nine audio files to various categories of samples (3,470 are clean, 1,055 are noisy, and the rest are highly degraded sound samples). They did not, however, mention if a laboratory has verified the data. Next, the authors used a crowdsourced database designated database-2, with 6,631 users; 235 users were declared COVID-19 positive. The authors suitably optimized the frequency range and the conversion scale of the recordings. The authors used adaptive synthetic sampling approach for imbalanced learning to create synthetic data. In their experiments, they reported accuracy of 0.86 (0.74) for database-2 (database-1) cough sounds.

In a similar fashion, Mouawad, Dubnov & Dubnov (2021) analyzed symbolic recurrence quantification measures derived from MFCC features to detect COVID-19 cases using cough sounds and speech. They used recurrence dynamics and variable Markov model on sustained vowel ‘ah’ recordings and showed that their model is robust for detecting the disease in sustained vowel utterances. The dataset was composed of 1,927 cough sound samples with only 32 sick patients and 1,488 speech records with only 20 sick patients. The authors ensured that the classification model was not biased towards the majority class and experimented with a wide range of data sampling techniques such as oversampling the minority class or under-sampling the majority class. They reported the following test results: an accuracy of 0.91 and an AUC of 0.84.

In addition to handcrafted features, other studies highlight the use of typical machine learning classifiers, such as Neural Networks (NNs), Support Vector Machine (SVM), Random Forest (RF), and Logistic Regression (LR). In Shimon et al. (2021), the authors used multiple different shallow learning techniques like SVM and RF. Their dataset was from non-publicly available crowdsourced data that took multiple types of recordings from people over multiple days. The type of recording used in this article was both cough sounds and the vowel ‘a’. In total, 1,296 cough recordings were used, and 428 “a” recordings were used. The experimenters used multiple different techniques to extract the audio feature, including the openSMILE toolkit and Librosa. After the classifiers classified each audio sample, the experimenters used simple majority voting to classify the patient as COVID-19 infected or non-COVID-19 infected. SVM showed the best result of the different classifiers with an accuracy of 0.78 and 0.74, an AUC of 0.64 and 0.60, a sensitivity of 0.95 and 0.90, and a specificity of 0.36 and 0.35 for ‘a’ and cough, respectively.

In Despotovic et al. (2021), the authors used wavelet scattering features and deep audio embeddings with multiple different shallow learning techniques. Their dataset used crowdsourced data with 1,103 participants where each produced two to five samples each. From these samples, the authors created a balanced dataset with a total of 496 cough samples. They used five-fold leave one out cross-validation for their experimental set-up. Boosting with the wavelet scattering features seemed to have the best overall performance with a 0.88 accuracy, a 0.87 sensitivity, and a 0.89 specificity. In Melek (2021), the authors used multiple different shallow learning architectures to classify cough as COVID-19 Positive or Negative. Among the different architectures are Polynomial-SVM, Linear-LDA, and Euclidean-kNN. They used a composition of two different datasets, 121 from Virufy and 59 from NoCoCoDa. Once combined, the dataset consisted of 107 positive samples and 73 negative samples. Unfortunately, the paper did not specify how they split their data, which limits the reproducibility of this model and limits interpretation of the results. Euclidean-kNN achieved their best results on a single cough with a 0.98 accuracy, 1.0 Specificity, 0.97 Sensitivity, and 0.99 AUC.

• Deep features (deep learning):

The authors also introduced a Deep Neural Network (DNN) to analyze cough sounds. In Nessiem et al. (2021), the authors explore the usage of deep learning models as a ubiquitous, low-cost, pre-testing method for detecting COVID-19 from audio recordings of breathing or coughing taken with mobile devices or *via* the web. First, they collected 1,427 audio files from a crowdsourced database. Then, they adapted an ensemble of three Convolutional Neural Networks that utilize breathing and coughing raw audio, spectrograms, and Mel-spectrograms to analyze if a speaker is infected with COVID-19 or not. In their experiments, they reported an unweighted average recall/sensitivity of 0.75, AUC of 0.81, and accuracy of 0.73 by ensembling NNs.

In Gokcen et al. (2021), the authors used a “Deep neural network” for the classification of COVID-19 infected individuals. However, the paper did not give an excellent description of their neural network, and from what was gathered, they used some dense net with neurons. However, it is clear that the paper had a sample size of 822 coughs from the MIT open dataset. This seemingly small neural network produced the following results: an accuracy of 0.79 and a recall of 0.75.

In Feng et al. (2021), the authors used an rNN to classify COVID-19 infected individuals from non-infected individuals. They used two different datasets, Coswara for the one they split into validation and training sets, the next is a small Virufy dataset for its final test set. Coswara consisted of 1,433 participants, which they used a 0.80, 0.20 split. The small Virufy test set consisted of 16 recordings from seven patients. Their model had an encouraging result for the training and validation set; however, when they tried it on the Virufy dataset, they could only get around a 0.81 accuracy with an AUC of 0.79.

In Coppock et al. (2021), the authors used a deep neural network on a crowdsourced dataset. This study used a “CIdeR” Convolutional Neural Network (CNN) which is based on the ResNet architecture to help classify in four tasks: (a) COVID positive and stratum healthy-no-symptoms (62 *vs* 245 subjects); (b) COVID-positive with COVID cough and stratum healthy-with-cough (23 *vs* 30 subjects); (c) COVID-positive with COVID cough and stratum asthma-with-cough (23 *vs* 19 subjects); and (d) COVID positive and COVID negative (62 *vs* 293 subjects). In total, this dataset consisted of 517 samples from 355 participants, and each sample was chopped up into segments, then used a voting mechanism with a mean average across the segments to break ties. Their respective AUCs are: (a) 0.83, (b) 0.57, (c) 0.91, and (d) 0.85.

In Lella & Pja (2021), the authors extended the work of Brown et al. (2020) (using the exact same dataset) using a Deep CNN with multi-feature channels and data augmentation. In contrast to previous work, their results were improved. It was, however, not mentioned whether the improvement was due to data augmentation, the use of the DCNN classifier, or the increase in the dataset’s size. These authors are also ambiguous in declaring whether their data is entirely non-laboratory confirmed, as they mention that users have been admitted ‘into the clinic.’ To classify between COVID-19 positive and negative cases, an accuracy of 0.95 was reported. No other metrics such as sensitivity and specificity were used. In Pahar et al. (2021), they used multiple different machine learning techniques on a crowdsourced publicly available dataset called the ‘Coswara’ dataset. It was composed of 92 (1,079) COVID-19 positive samples (negative samples). The authors generated synthetic COVID-19 samples to build dataset balanced. Of many classifiers (*e.g*., LR, LSTM, CNN and ResNet50), ResNet50 achieved the best-reported accuracy (0.95). However, throughout the entire reported results, sensitivity remained in the lower nineties (percentage-wise). Later, the researchers also tested their model on a clinically validated dataset called Sarcos after training on the Coswara dataset. Their best performing model was a combination of LTSM + SFS which had an accuracy of 0.9291, AUC of 0.938, sensitivity of 0.91, and a specificity of 0.96.

In addition, the authors have found that biomarkers are important features. In Laguarta, Hueto & Subirana (2020), their tool helped extract and leverage acoustic biomarker features to help pre-screen COVID-19 recordings. The dataset was composed of 475 laboratory-confirmed COVID-19 infected patients, 962 doctor assessments, and 1,223 personal assessments. This study used under-sampling to pull coughs from a group of ‘hundreds of thousands’ of non-infected COVID-19 people to balance the dataset with 224 official tests, 523 personal assessments, and 1,913 personal assessments. However, this study does not thoroughly explain what a ‘personal assessment’ is. However, this study does explain that a diagnostic test must have been complete within seven days of of collection of the sample, with symptom onset no later than 20 days. In total, 4,256 cough samples were used for training and 1,064 for validation. The CNN incorporates multiple biomarker feature models. These biomarkers included muscular degradation, vocal cords, sentiment, and lung and respiratory tract. They achieved a forced cough COVID-19 screening accuracy of 0.97, an AUC of 0.97, a sensitivity of 0.98, and a specificity of 0.94. Furthermore, asymptomatic patients achieved a sensitivity of 1.0 with 0.83 specificity. This work admits that both the inclusion of doctor assessments and personal assessments hurts the generalizability of their model, and are conducting future tests. Since the results are high, their methods of biomarker use should be further explored. In Jayachitra et al. (2021), the authors used a deep neural network architecture. The paper proposed a RNN that would fuse each mode of input’s predictions and add the symptoms to the mix of fusions. The dataset used a mixture of audio and radiological pictures to get a yes or no for COVID-19 infection. After gathering many datasets, their cough sample size consisted of 153 positive samples and 348 negative samples. Again, artificial data was used in their sample size. Their testing procedure did not include a separate test set, so there is a potential bias toward the dataset with the .80 .20 split. However, they report that their multi-modal dataset scored 1.0 across the board on all metrics with just cough at 0.97 accuracy, 0.97 precision, and 0.94 recall. As the model is still progress, it may not be generalized well to the population.

#### Studies on laboratory confirmed datasets

In Table 2, we observe the use of CNN and/or DNN as well as ensemble learning to detect COVID-19 in the cough sounds of human subjects. Based on the authors’ reports, we categorize their studies with laboratory confirmed datasets. In what follows, we discuss CNN-based works as well as a new paradigm of physiological impact.

CNN has been popularly used to develop AI-guided tools across many different computer vision tasks, and COVID-19 screening using cough sounds is no exception. In Imran et al. (2020), the app called ‘AI4COVID-19’ recorded three seconds of sound and provided results in less than 2 min. This app used a CNN to identify cough sounds. In their first test, they employed Deep Transfer Learning-based Multi-Class classifier (DTL-MC) CNN with multiple output classifiers for COVID-19 and three other diseases. The second used a Classical Machine Learning-based Multi-Class classifier (CML-MC) to judge whether the first one suffered from over-fitting issue. The last test employed a Deep Transfer Learning-based Binary Class classifier (DTL-BC) (similar to the first one), but it was limited to a binary output (yes/no) for a possible COVID-19 infection. For training, they used 1,838 cough sounds and 3,597 non-cough environmental sounds, and for testing, they used 96 bronchitis, 130 pertusses, 70 COVID-19, and 247 normal cough samples. The authors did not mention whether they were laboratory-confirmed, but they referred to the people that provided the samples as “patients,” so we assume these samples were collected in a hospital/clinical environment, and thus are laboratory confirmed. The DTL-BC classifier in this study provided the best overall accuracy at 0.93 in differentiating COVID-19 from non-COVID-19 cough sounds and had a sensitivity of 0.96, and specificity of 0.91.

Another form of NN called Recurrent Neural Network (RNN) was used in the literature. In Pinkas et al. (2020), the authors collected self-recordings from phones (vocal utterances, speech and cough sound), and used RNN to produce specialized sub-models for the SARS-CoV-2 classification. From 29 laboratory-confirmed COVID-19 patients and 59 negative control subjects, 235 samples were used for training the model and 57 samples for testing. An ensemble stacking fused the predictions of the sub-models and pre-training, bootstrapping and regularization techniques were used to prevent over-fitting. They reported an accuracy of 0.79 (with a corresponding sensitivity of 0.79) based on leave-one-out validation protocol.

Cough sounds may not be sufficient to completely analyze COVID-19 positive cases, and physiological impact is another important data type that could help better analyze. In Lonini et al. (2021), the authors introduced a novel paradigm based on recording the physiological responses elicited by a short sequence of 2-min activities (physical activity, cardio-respiratory function and cough sounds). While validating the data, they employed a novel body-conforming soft wearable sensor placed on the suprasternal notch to capture physical activity data and cardio-respiratory function. Combining these features on snapshots from 19 COVID-19 positive and 14 healthy cases provided an AUC of 0.94 as compared to 0.64 (with only forced cough sounds).

### Research articles outside of PubMed and Web of Science

In order not to miss up-to-date experimental-based research works, we used the exact same keywords in search engines: ACM, IEEE, Springer, and Elsevier. Using the exact same inclusion criteria, these articles are summarized in Table 3, and their respective results on laboratory confirmed data.

**Table 3:** Cough sounds (including other data type) for COVID-19 screening performance in terms of Accuracy (ACC), Area Under the Curve (AUC), Sensitivity (SEN) and Specificity (SPEC).

		Performance			
Authors (year)	Data type (sample size)	ACC	AUC	SEN	SPEC
Han et al. (2021)	Multiple data types* (828)	–	0.79	0.62	0.74
Brown et al. (2020)	Multiple data types* (430)	–	0.80	0.69	–
Vrindavanam et al. (2021)	Cough sounds (150)	0.84	0.88	0.81	–
Anupam et al. (2021)	Cough sounds (640)	0.97	0.98	0.97	–
Bansal, Pahwa & Kannan (2020)	Cough sounds (500)	0.70	–	0.81	–
Grant, McLane & West (2021)	Multiple data types* (2,239)	–	0.68	–	–
Nessiem et al. (2021)	Multiple data types* (1,427)	0.73	0.81	0.75	–
Khriji et al. (2021)	Multiple data types* (3,718)	0.80	–	0.78	–
Pal & Sankarasubbu (2021)	Multiple data types* (328)	0.95	–	0.90	0.97
Wei et al. (2020)	Cough sounds (1,283)	0.76	–	0.99	0.95
Hassan, Shahin & Alsabek (2020)	Cough sounds (80)	0.97	0.97	0.96	–
	Breathing sounds (80)	0.98	0.98	0.98	–
	Voice sounds (80)	0.88	0.84	0.91	–
Andreu-Perez et al. (2021)	Cough sounds (8,380)	–	0.99	0.96	0.96

**Note:**

* Multiple data types, including cough sound.

Han et al. (2021) proposed a voice-based framework to automatically detect COVID-19 positive cases and evaluated the performance on a subset of data crowdsourced from the ‘COVID-19 Sound App’. The authors used InterSpeech 09 Computational Paralinguistics Challenge (COMPARE) set, openSMILE toolkit, MFCCs features, and used SVM with linear kernel as the classifier. While using the app, users got asked to record information in the app by submitting their breathing, coughing, and voice samples along with reported symptoms, if any, and provide some basic demographic and medical information. On 828 samples (326 COVID-19 positives and 502 COVID-19 negatives), the authors reported an AUC of 0.79 with a sensitivity of 0.68 and a specificity of 0.74.

In Brown et al. (2020), the authors used data analysis over a large-scale crowdsourced dataset, however some of their data seems to be laboratory confirmed as they asked their patients whether or not they were in a hospital. Furthermore, the dataset consists of 154 cough and breathing sounds from self-reported COVID-19 infected users, of which 54 report a dry cough. Also, the control groups of the experiment consisted of 298 non-COVID-19 users, 32 non-COVID-19 users with a cough, and 20 non-COVID-19 users with asthma and cough. In all cases, they used an 80/20 split for training and analysis and under-sampled the majority for training. They tested classifiers, such as LR, Gradient Boosting Trees, SVM (with a radial basis function kernel). They reported binary classification tasks for: (a) differentiating COVID-19 users from non-COVID-19 users; (b) differentiating COVID-19 users with a cough from non-COVID-19 users with a cough; and (c) differentiating COVID-19 users with cough from non-COVID-19 user with asthma and cough. In their results, the authors found an AUC of 0.80 across all tasks and a sensitivity of 0.69.

Similarly, in Vrindavanam et al. (2021), the authors presented an approach to classify audio samples between COVID-19 patient and a healthy person by taking LR, SVM and RF classifiers into account. Their dataset was composed of 150 cough audio samples, of which 54 were COVID-19 positive, and the study did not state whether they were laboratory confirmed. In their test, SVM performed better than LR and RF on all performance metrics and reported an accuracy of 0.84 (with AUC of 0.88). As the dataset size was small, the results could possibly be biased, which was not mentioned in their article.

In Anupam et al. (2021), the authors analyzed COVID-19 cough sounds to detect COVID-19. The dataset is composed of 640 cough samples (source: Coswara database): 160 infected and 480 healthy cases. Interestingly, other papers written around this paper had almost double the number of cough samples. However, the paper did not specify what technique they used for eliminating assumingly half of the dataset, along with any other pre-processing techniques they may have used. For classification, the authors used shallow-based machine learning classifiers such as LR, KNN, SVM, and decision tree algorithms. The SVM classifier performed the best of all: AUC of 0.98, accuracy of 0.99, sensitivity of 0.97, precision of 0.99, and F1 score of 0.98.

In Mohammed et al. (2021), the authors developed a robust classifier for a COVID-19 pre-screening model from crowdsourced cough sound data. While detecting COVID-19 from sound datasets, the authors faced two main challenges. The first challenge being a variable number of coughs in each recording, and the second is the low number of COVID-19 positive cases compared to healthy coughs in the data. In total, they were able to obtain 8,886 cough samples which they then used under-sampling the majority to create a balanced dataset of 1,276 cough samples. After obtaining their balanced dataset, they used a VGG16 to extract the audio features such as Mel-spectrograms, MFCCs, spectrograms, and even used the raw audio data to obtain 25,088 feature vectors per audio input. Afterward, they used two separate training pipelines with ensemble learning, one with shallow-based learning such as LR, SVM, and K-Nearest Neighbor, and the second with ensemble learning of three different CNNs, one CNN built from scratch along with two pre-trained VGG models. Their method illustrated a respectable performance using an ensemble model on the testing dataset with AUC of 0.77, precision of 0.80, recall of 0.71, F1 score of 0.75, Kappa of 0.53, and an accuracy of 0.77.

In Bansal, Pahwa & Kannan (2020), the authors proposed a CNN-based audio classifier using an open cough dataset. They used a manually labeled dataset that was composed of two categories: COVID-19 and non-COVID. They proposed two different approaches: one is based on MFCC features and another used spectrogram images for CNN network. First, they took a dataset of 911 cough sounds, where 871 are from YouTube videos and 40 are from audio files. Afterward, they pared that down to 500 audio samples after labeling. The authors found that the MFCC approach produced 0.71 test accuracy, 0.81 sensitivity, 0.61 precision, and 0.69 F1 score. These results were better than the spectrogram-based approach. It would be interesting to see the results of this paper’s methods along with mel-spectrograms as well.

Grant, McLane & West (2021) developed a method that can be applied to analyze sounds to detect COVID-19 on a crowd-sourced data with sound recordings and these were self-identified. They took a total of 1,040 (78 COVID-19 positive) cough samples and 1,199 (81 COVID-19 positive) speech and breathing samples. MFCCs and relative spectra perceptual linear prediction features were evaluated independently with two different classifiers: DNN and RF. The following AUCs were reported: 0.6836 from cough sounds (DNN classifier), 0.79 from speech sound (RF classifier), and 0.76 from breathing sound (DNN classifier).

Khriji et al. (2021) proposed a deep Long Term Short (LSTM) technique to detect COVID-19 infections from cough, breath and sneeze signals *via* smartphones or wearable sensors. The dataset was composed of audio signals like cough, sneeze and breath. Furthermore, this dataset was partitioned further into three subsets, including a training set (sick (1,435) + not sick (2,283)), a validation set (sick (468) + not sick (753)), and a test set (sick (642) + not sick (1,012)). The authors, however, did not mention whether dataset was laboratory confirmed. The authors reported an accuracy of 0.80 and a sensitivity of 0.78.

In Pal & Sankarasubbu (2021), the authors evaluated their model using a medical dataset containing symptoms and demographic data of 30,000 audio segments. They extracted 328 cough sounds from 150 patients with four cough classes (COVID-19, Asthma, Bronchitis and Healthy). They used a CNN to classify cough sounds, and the study showed that their model captured many robust features of cough sounds to distinguish between COVID-19 coughs and several types of non-COVID-19 coughs. The authors reported an accuracy of 0.95, a sensitivity of 0.90, a specificity of 0.97, F1 score of 0.90, and precision of 0.91.

In Wei et al. (2020), the authors analyzed a real-time robot-based tool to evaluate risk level due to COVID-19 infection. They used real-time speech analysis, temperature, keyword, cough, and other functions to convert live audio into structured data. The authors collected a dataset of 1,283 speech recordings *via* human-robot conversations from 184 people for the test evaluation. Of all, 392 segments from 64 people were laboratory-confirmed COVID-19 infected. The remainder of the samples were healthy individuals with a history of smoking, acute bronchitis, chronic pharyngitis, children with pertussis and healthy people with no smoking history. For cough detection, using a CNN, they reported an accuracy of 0.76, while their sensitivity was high (0.99).

Similarly, Hassan, Shahin & Alsabek (2020) studied early screening and diagnoses of COVID-19 patients by using RNN and leveraged its significant architecture to discover the acoustic features of cough, breathing and voice of the patients. In their study, 60 healthy and 20 COVID-19 infected patients were asked to record three separate samples: cough, breath and voice sounds. The COVID-19 infected patient samples got collected from hospitals in the UAE. With data split, 70/30 (train/test), they achieved the following accuracies: 0.97 for cough sounds, 0.98 for breathing sounds, and 0.88 for voice sounds.

DNN is no exception in cough sound analysis in detecting COVID-19. In Andreu-Perez et al. (2021), the authors used empirical mode decomposition with the tensor of speech features and a Deep Artificial Neural Network (ANN) to detect cough of COVID-19 patients. Of all, 8,380 samples of cough sounds were collected *via* a web app called ‘Cough Detect’ to record the coughs anonymously; 2,339 of the samples are from patients with confirmed qRT-PCR laboratory tests for the COVID-19 infection. The authors reported promising results: AUC of 0.99, sensitivity of 0.96, and specificity of 0.96.

## Discussion

In this section, we summarize our observations based on the current state-of-the-art works by considering cough sound as primary data. Our discussion is based on cough sound analysis *via* AI-guided tools for COVID-19 screening (*ref*. “AI-guided tools for COVID-19 screening using cough sounds”).

(a) Cough symptoms present themselves at a high rate relative to other symptoms in the vast majority of studies (*ref*. “Introduction”). It stands to reason that this will also translate well into the population at large, and the majority of the general population would present a cough if infected by COVID-19. In addition to other clinical tests, cough tests could potentially help build clinical decisions for COVID-19 positive. In other words, cough is not the only reliable metric, it is however comparable to others, such as fever. AI-guided tools (*ref*. “AI-guided tools for COVID-19 screening using cough sounds”) can analyze cough sounds and help detect COVID-19 at a very high rate regardless of cough diagnosis. This fact, along with affordability and accessibility, could make AI-guided cough screening a first-line defense against COVID-19 and similar infectious outbreaks, especially in resource-constrained regions.

(b) Following the state-of-the-art methods for screening COVID-19 infections (*ref*. “AI-guided tools for COVID-19 screening using cough sounds”), we observed a clear distinction between two types of datasets: with and without laboratory confirmation (see Tables 1 and 2).

On laboratory confirmed datasets, AI-guided tools performed better as compared to non-laboratory confirmed data in the vast majority of reviewed articles. Also, the laboratory confirmed data could potentially provide convincing results, as they were annotated by experts. Machine learning algorithms require enough training data (with all possible positive cases). Moreover, AI-guided tools trained with fairly large amounts of data can be accurately used to screen COVID-19 human subjects from a forced cough regardless of the dataset, even if performance degrades slightly on non-laboratory confirmed data. In all cases, importantly, the authors did not mention whether their AI-guided tools are externally validated and reproducible (Topol, 2020). Moreover, most studies with high metrics in the non-laboratory confirmed sections have serious deficiencies in the reporting of their methods, or their model is admittedly biased.

(c) Integrating other data types such as sneezing, respiration, speech, throat clearing, wheezing, and breathing can help build a better decision-making process (see Tables 1 and 2).

(d) After reviewing the use of shallow learning and deep learning (*ref*. “AI-guided tools for COVID-19 screening using cough sounds”), we observed that the use of deep features could open the genericity of the model rather than relying on prior knowledge.

(e) In designing AI-guided tools, we observed the use of additional features, such as biomarkers and clinical data (Laguarta, Hueto & Subirana, 2020). These features get ensembled into the CNN that utilizes the standard features extracted from audio recordings. Such a feature integration could potentially drive future works. Introducing new paradigms (*e.g*., biomarkers) could help better analyze COVID-19.

## Conclusion

In this article, we have systematically reviewed state-of-the-art works in both years 2020 and 2021 by taking into account AI-guided tools to analyze cough sounds for COVID-19 screening. Clinically, we have found that cough (*via* the use of cough presentation rate in *ref*. “Introduction”) is considered as one of the primary symptoms in severe and non-severe infections alike. COVID-19 screening (*ref*. “AI-guided tools for COVID-19 screening using cough sounds”) using cough sounds is found to be potentially a cheap, effective, and available alternative to help people decide to quarantine or get tested. In other words, as cough is ubiquitously presented among varying populations, it is possible to develop AI-guided tools with high accuracy to do mass screening using cough sounds. This method of screening deserves to get more thoroughly investigated and developed into production *via* a mobile app.

Integrating other data types, such as sneezing, respiration, speech, throat clearing, wheezing, breathing, biomarkers and clinical data, can help build a better decision-making process; we will extend our work by implementing ensemble DNNs within a multimodal learning mechanism.

## Additional Information and Declarations

### Data Availability

The following information was supplied regarding data availability: PubMed and Web of Science were used for this literature review; there is no raw data.
